# Epidemiology of pneumonia in the pre-pneumococcal conjugate vaccine era in children 2-59 months of age, in Ulaanbaatar, Mongolia, 2015-2016

**DOI:** 10.1371/journal.pone.0222423

**Published:** 2019-09-11

**Authors:** Claire von Mollendorf, Sophie La Vincente, Mukhchuluun Ulziibayar, Bujinlkham Suuri, Dashtseren Luvsantseren, Dorj Narangerel, John de Campo, Margaret de Campo, Cattram Nguyen, Sodbayar Demberelsuren, Tuya Mungun, E. Kim Mulholland

**Affiliations:** 1 New Vaccines, Murdoch Children’s Research Institute, Royal Children’s Hospital, Parkville, Vic., Australia; 2 Department of Paediatrics, The University of Melbourne, Parkville, Australia; 3 National Center of Communicable Diseases (NCCD), Ministry of Health, Ulaanbaatar, Mongolia; 4 Ministry of Health, Ulaanbaatar, Mongolia; 5 Department of Radiology, The University of Melbourne, Parkville, Australia; 6 Expanded Programme on Immunization, World Health Organization, Ulaanbaatar, Mongolia; 7 Department of Infectious Disease Epidemiology, London School of Hygiene and Tropical Medicine, London, England, United Kingdom; MAHSA University, MALAYSIA

## Abstract

**Background:**

Respiratory diseases, including pneumonia, are the second largest cause of under-five mortality in Mongolia and the most common cause of childhood hospitalization. However information regarding the contribution of *Streptococcus pneumoniae* to pneumonia causation in Mongolia is limited. We aimed to describe the epidemiology of hospitalized children aged 2–59 months with pneumonia, enrolled into a surveillance program in the period prior to pneumococcal conjugate vaccine (PCV) introduction, in Mongolia.

**Methods:**

An expanded pneumonia surveillance program enrolled children, who met the surveillance case definition, at participating hospitals, between April 2015 and May 2016. Cumulative incidence rates were calculated by district for all pneumonia endpoints using district specific denominators from the Mongolian Health Department census for 2016. Socio-economic and disease-associated factors were compared between districts using chi-squared tests.

**Results:**

A total of 4318 eligible children with pneumonia were enrolled over the 14 month period. Overall the incidence for all-cause pneumonia in children aged 12–59 months was 31.8 per 1000 population; children aged 2–11 months had an almost four-fold higher incidence than children aged 12–59 months.

Differences were found between districts with regards to housing type, fuel used for cooking, hospital admission practices and the proportions of severe and primary endpoint pneumonia.

**Discussion:**

This study shows a high burden of pneumonia in children aged 2–59 months in Mongolia prior to PCV introduction. Rates differed somewhat by district and age group and were influenced by a number of socio-economic factors. It will be important to consider these differences and risk factors when assessing the impact of PCV introduction.

## Introduction

Ninety-five percent of all deaths from acute lower respiratory tract infections occur in low- and middle-income countries [1] with more than three quarters of childhood deaths from pneumonia occurring in the first two years of life [2]. Respiratory diseases, including pneumonia, are the second largest cause of infant and under-five mortality in Mongolia and the most common cause of childhood hospitalization [3]. This is not surprising considering the extremely cold winter temperatures and high levels of air pollution in urban areas [4]. In 2015, the under-5 mortality rate in Mongolia was reported as 18.3/1000 live births [3] with all respiratory disease accounting for 15% of infant deaths in children under 5 years.

Pneumonia is caused by a number of infectious agents; the most common causes being *Streptococcus pneumoniae*, *Haemophilus influenzae* type b and respiratory syncytial virus.

Information regarding the proportional contribution of various etiological agents to pneumonia in Mongolia is limited. In 2000, it was estimated that globally around 800,000 children <5 years of age died from pneumococcal disease, with 47,300 of these deaths occurring in the Western Pacific region [5].

Studies have identified a number of risk factors for childhood pneumonia [6]. Definite risk factors include malnutrition, non-exclusive breastfeeding during the first 4 months of life, indoor air pollution and crowding; likely risk factors include parental smoking and concomitant diseases; and possible risk factors include low maternal education, day-care attendance and outdoor air pollution.

Mongolia introduced the 13-valent pneumococcal conjugate vaccine (PCV13) from the 1^st^ June 2016 accompanied by an enhanced hospital-based pneumonia surveillance program [7]. In this paper we present the baseline incidence rates and characteristics of children 2–59 months of age with pneumonia, who were enrolled into the surveillance program prior to PCV13 introduction (April 2015-May 2016) in Mongolia. We also describe and compare the characteristics of districts identified for different phases of PCV13 introduction. There is a need to document the burden of pneumonia in the pre-PCV era due to a lack of data in the region and to assist in the interpretation of changes that occur following vaccine introduction.

## Methods

### Study setting

PCV13 was introduced in a phased manner into the routine immunization program at 2, 4 and 9 months of age, with a catch-up campaign to 24 months of age. As previously described four districts of the capital city, Ulaanbaatar, were planned for initial introduction [7]. The two “phase 1” districts (Songinokhairkhan and Sükhbaatar) had PCV introduced from June 2016, while the “phase 2” districts were staggered (Bayanzürkh had vaccine introduced from 1^st^ July 2017 and Chingeltei in March 2018). Hospital-based pneumonia surveillance for children 2–59 months was expanded in these four districts from April 2015. It was modified from the routine World Health Organization Invasive Bacterial Vaccine Preventable Disease (WHO IB-VPD) Surveillance Network that was initiated in 2007 [8].

### Study population and design

Cases included children 2–59 months of age admitted with pneumonia at one of the four participating district hospitals, or the tertiary hospital if they resided in one of the relevant districts. Cases who met the surveillance case definition were enrolled from April 2015 to May 2016. A retrospective chart review was also conducted to ensure that all cases of pneumonia were included in the surveillance program. Protocol details have been previously published [7].

The study case report form (CRF) was based on the pre-existing WHO IB-VPD CRF. The CRF included information on demographic variables, presenting symptoms and signs, previous medication, immunization history, oxygen saturation and therapy and treatment received. In addition, a risk factor questionnaire was also completed. Blood cultures were collected for suspected pneumonia and sepsis cases and nasopharyngeal swabs were collected, handled and transported according to WHO recommended methods [9] for all enrolled cases who consented.

### Case definitions and study outcomes

The pneumonia study endpoints included in our analysis were WHO-defined primary endpoint pneumonia (PEP) [10] as the primary outcome, and severe pneumonia and very severe pneumonia as the secondary outcomes (Table 1). Other secondary outcomes, related to pneumonia and carriage, that are part of the broader vaccine impact evaluation are described in a previous publication [7] and are not discussed.

**Table 1:** Case definition and pneumonia endpoint definitions used in the pneumonia surveillance program.

Category	Criteria
**Surveillance case definition**	
All pneumonia	Cough or difficulty breathing, with one of the following: • an elevated respiratory rate (≥50 bpm for all ages) • oxygen saturation <90%; • lower chest wall indrawing
**Pneumonia endpoint definitions**	
WHO-defined primary endpoint pneumonia (PEP)	End-point consolidation (dense or fluffy opacity that occupies a portion or whole of a lobe or the entire lung that may or may not contain air bronchograms) OR pleural effusion that is in the lateral pleural space and associated with pulmonary parenchymal infiltrate or if the effusion obliterated enough of the hemithorax to obscure an opacity.
Severe pneumonia (IMCI 2005 criteria [11])	Cough or difficulty breathing and tachypnoea plus • Lower chest indrawing OR • General danger sign (inability to breastfeed or drink, persistent vomiting, lethargy or reduced level of consciousness, convulsions or severe malnutrition) OR • Oxygen saturation < 90% or central cyanosis
Very severe pneumonia	Severe pneumonia with one or more of the following: • ICU admission/supplementary oxygen used • hypoxia (O2 sat < 90%)^a^ • death • persistent signs of severe illness post-discharge • empyema

^a^ Only relevant for severe pneumonia cases classified based on chest indrawing or general danger sign criteria.

### Statistical analysis

We calculated cumulative incidence rates (IRs) stratified by age group and district for all pneumonia endpoints using district specific denominators from the Mongolian Health Department census for 2016. As there were no separate population data for infants <2 months of age, we assumed this age group included 2/12 of the population <1 year of age. This calculated number was subtracted from the population denominator for children aged <1 year to estimate a denominator for the 2–11 month age group. Confidence intervals for incidence estimates were calculated using a Poisson distribution.

As chest X-rays (CXRs) were missing for retrospectively collected data, we used multiple imputation to estimate the presence or absence of PEP for the cases who did not have CXRs done. The predictors for the multiple imputation model (S1 Text) were based on those factors that differed significantly between patients with and without positive CXR findings (S1 Table). To determine whether there were differences between pneumonia cases who had PEP detected on CXR and those who had no abnormalities detected on CXR we compared characteristics and risk factors between these two groups using logistic regression. Variables with a p-value <0.20 on univariate analysis were included in the model for multivariable analysis to estimate adjusted odds ratios. All non-significant factors (assessed at p-value >0.05) were dropped from the multivariable model employing stepwise regression starting with predictors with the highest p-values.

To determine if there were differences between phase 1 and phase 2 districts we compared socio-economic and disease-associated factors in children resident in these districts using a chi-squared test. Statistical analysis was implemented using Stata version 14.2 and version 15.1 (StataCorp LP, College Station, TX, USA).

### Ethical considerations

The protocol was approved by the Human Research Ethics committees of the Mongolian Ministry of Health and Sports, Ulaanbaatar, the Royal Children’s Hospital, Melbourne (HREC 33203A), and the Ethics Review Committee of the World Health Organization Western Pacific Regional Office. Written informed consent was obtained from the caregivers of all participants.

## Results

**Fig 1:**
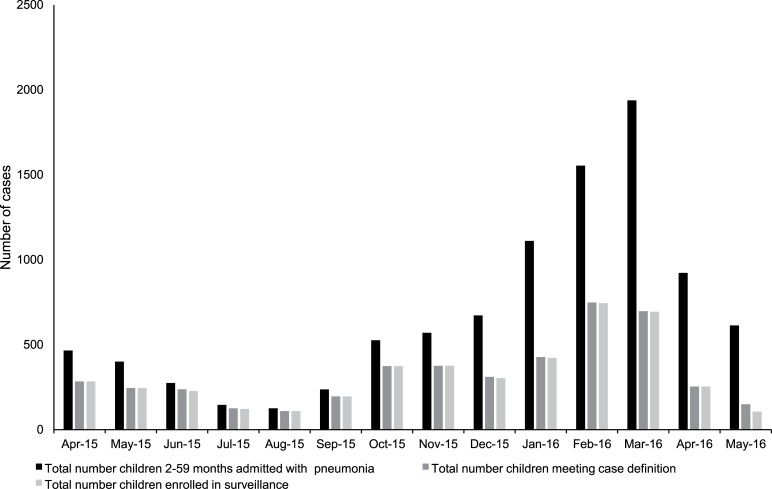
Total pneumonia admissions and children 2–59 months screened and enrolled into the surveillance program, Mongolia, April 2015-May 2016. *Numbers of children meeting the case definition were not available for April and May 2015, so they were assumed to be equal to enrolments numbers based on experience from other months.

From a total of 9559 children who were admitted with any respiratory disease between April 2015 and May 2016, and were screened for the study, approximately half (4540, 47%) met the pneumonia surveillance case definition and 4460 children were enrolled (Fig 1). Of those children enrolled, 142 were found to be ineligible (17 not age eligible and 125 who had not lived in the target districts for at least three months), resulting in 4318 children 2–59 months of age included in the analysis. Of the included children, 24% (1056/4318) were identified by retrospective chart review. In children with a known outcome (n = 3265), <1% were reported to have died during their admission (n = 16).

Pneumonia admissions showed a seasonal pattern (Fig 1) with the highest number of admissions and enrolments over the winter months (November-March) and the lowest numbers in summer (June-August). Approximately three quarters (3162/4318, 73%) of all pneumonia admissions in the enhanced surveillance program were in children 2–23 months of age.

### Pneumonia incidence rates

Overall the incidence for all-cause pneumonia in children 2–59 months of age was 31.8 (95% confidence interval [CI] 30.9–32.8) per 1000 population (Table 2). Children aged 2–11 months had an almost four-fold higher incidence (83.7 [95% CI 79.9–87.8] per 1000 population) of all-cause pneumonia than children aged 12–59 months (22.4 [95% CI 21.5–23.3] per 1000 population). A similar difference was seen in PEP (9.2 [95% CI 7.9–10.6] per 1000 for 2–11 months compared with 2.5 [95% CI 2.2–2.8] per 1000 for 12–59 months). The incidence for severe pneumonia was around 19 per 1000 population and very severe pneumonia 8 per 1000 population for all children aged 2–59 months. Incidence rates differed between the four districts and were generally higher in the Songinokhairkhan and Sükhbaatar (“Phase 1”) Districts compared with the Bayanzürkh and Chingeltei (“Phase 2”) Districts in all age groups (Table 2).

**Table 2:** Incidence rates for pneumonia by category, age and district, in children 2–59 months of age in Mongolia, April 2015-May 2016 (per 1000 population).

	Incidence rate (95% CI) All districts	Incidence rate (95% CI) Phase 1 districts	Incidence rate (95% CI) Phase 2 districts	Incidence rate (95% CI) Songinokhair-khan District	Incidence rate (95% CI) Sükhbaatar District	Incidence rate (95% CI) Bayanzürkh District	Incidence rate (95% CI) Chingeltei District
**All pneumonia cases**							
**2–11 months**	83.7 (79.9–87.8)	90.9 (85.2–96.9)	76.6 (71.3–82.0)	77.1 (71.0–83.6)	128.5 (115.6–142.5)	84.9 (78.1–92.1)	61.0 (53.2–69.6)
**12–59 months**	22.4 (21.5–23.3)	25.1 (23.8–26.5)	19.8 (18.6–20.9)	21.0 (19.6–22.5)	35.0 (32.3–38.0)	18.7 (17.4–20.1)	22.0 (19.9–24.2)
**2–59 months**	31.8 (30.9–32.8)	35.4 (34.0–36.9)	28.3 (27.1–29.6)	30.0 (28.5–31.6)	48.5 (45.5–51.7)	28.3 (26.8–29.9)	28.4 (26.2–30.7)
**Primary endpoint pneumonia cases**							
**2–11 months**	9.2 (7.9–10.6)	11.5 (9.5–13.8)	6.9 (5.4–8.7)	11.8 (9.5–14.5)	10.7 (7.2–15.3)	8.1 (6.1–10.5)	4.7 (2.7–7.5)
**12–59 months**	2.5 (2.2–2.8)	3.3 (2.9–3.8)	1.8 (1.4–2.1)	2.9 (2.4–3.5)	4.3 (3.3–5.4)	1.3 (1.0–1.8)	2.6 (1.9–3.5)
**2–59 months**	3.6 (3.2–3.9)	4.6 (4.1–5.1)	2.5 (2.2–2.9)	4.4 (3.8–5.0)	5.2 (4.2–6.3)	2.3 (1.9–2.8)	3.0 (2.3–3.8)
**Severe pneumonia cases**							
**2–11 months**	58.9 (55.6–62.2)	66.4 (61.6–71.6)	51.2 (47.0–55.8)	59.3 (53.9–65.0)	86.0 (75.5–97.6)	61.0 (55.3–67.2)	32.9 (27.2–39.3)
**12–59 months**	12.0 (11.3–12.6)	13.3 (12.4–14.3)	10.7 (9.8–11.5)	11.6 (10.6–12.7)	17.3 (15.4–19.5)	11.4 (10.4–12.5)	9.0 (7.7–10.5)
**2–59 months**	19.2 (18.4–19.9)	21.6 (20.5–22.8)	16.8 (15.8–17.8)	19.3 (18.1–20.6)	27.3 (25.0–29.7)	18.6 (17.4–19.9)	12.9 (11.4–14.5)
**Very severe pneumonia cases**							
**2–11 months**	26.6 (24.5–28.9)	26.6 (23.5–29.9)	26.7 (23.6–30.0)	26.5 (22.9–30.4)	27.1 (21.4–34.0)	32.2 (28.1–36.8)	16.0 (12.2–20.7)
**12–59 months**	4.8 (4.4–5.3)	5.2 (4.7–5.9)	4.4 (3.9–5.0)	4.3 (3.7–5.0)	4.8 (3.8–5.9)	5.7 (4.9–6.4)	4.4 (3.5–5.4)
**2–59 months**	8.2 (7.7–8.7)	8.5 (7.8–9.2)	7.9 (7.2–8.6)	7.9 (7.1–8.7)	8.0 (6.8–9.3)	9.5 (8.7–10.4)	6.3 (5.3–7.4)

CI = confidence interval; Phase 1 districts = Songinokhairkhan District and Sükhbaatar District; Phase 2 Districts = Bayanzürkh District and Chingeltei District

### Characteristics of children living in different districts of Ulaanbaatar

Just over half of pneumonia surveillance patients were enrolled in the phase 1 districts of Ulaanbaatar (2364/4318, 55%). Risk factor questionnaires could not be collected for the 1056 cases enrolled retrospectively by chart review. Risk factor questionnaires were however undertaken for 95% (3088/3262) of cases prospectively enrolled in the surveillance. Among these cases, there were some baseline differences observed in the characteristics of enrolled children residing in phase 1 and phase 2 districts (Table 3). In phase 1 districts 63% (1496/2364) of pneumonia admissions were enrolled during the cold season (November to March inclusive), while in phase 2 districts only half of participants (53%, 1038/1954, p<0.001) were enrolled during this period. In phase 1 districts children were more likely to live in informal housing (41%, 680/1643) and households that used coal for cooking (69%, 1126/1642), compared with phase 2 districts (34%, 495/1445 and 56%, 805/1440 respectively). There were also differences in hospital admission practices, including length of stay, antibiotics and oxygen supplementation, and the proportions of severe pneumonia and PEP between the two districts.

**Table 3:** Characteristics of children 2–59 months of age living in the Phase 1 (Songinokhairkhan and Sükhbaatar) and Phase 2 (Bayanzürkh and Chingeltei) districts prior to PCV13 introduction in Mongolia, April 2015 to May 2016 (n = 4318).

Category	Sub-category	Case numbers n/N (%)		Chi squared p-value
		Phase 1 districts (SK/SB^a^)	Phase 2 districts (CHD/BZ^a^)	
**Sex**	**Female**	1066/2364 (45)	887/1954 (45)	
	**Male**	1298/2364 (55)	1067/1954 (55)	0.84
**Age**	**2–11 months**	948/2364 (74)	798/1954 (73)	
	**12–59 months**	1416/2364 (26)	1156/1954 (27)	0.54
**Season**^**b**^	**Cold season**	1496/2364 (63)	1038/1954 (53)	<0.001
	**Warm season**	868/2364 (37)	916/1954 (47)	
**Primary caregiver**	**Parent**	1474/1643 (90)	1319/1445 (91)	0.14
	**Not parent**	169/1643 (10)	126/1445 (9)	
**Mothers education**	**Tertiary**	822/1635 (50)	772/1431 (54)	0.04
	**Primary/Secondary**	813/1635 (50)	659/1431 (46)	
**Number of siblings**	**Any siblings**	558/1639 (34)	369/1440 (26)	<0.001
	**No siblings**	1081/1639 (66)	1071/1440 (74)	
**Crowding (People/room)**	**>3**	491/1623 (30)	355/1413 (25)	0.002
	**< = 3**	1132/1623 (70)	1058/1413 (75)	
**Smokers in the home**	**Yes**	736/1642 (45)	584/1443 (40)	0.02
	**No**	906/1642 (55)	859/1443 (60)	
**Fuel for cooking**	**Wood/Coal**	1126/1642 (69)	805/1440 (56)	<0.001
	**Electricity/gas**	516/1642 (31)	635/1440 (44)	
**Housing type**	**Informal**	680/1643 (41)	495/1445 (34)	<0.001
	**Formal**	963/1643 (59)	950/1445 (66)	
**Khoro (subdistrict) type**^**c**^	**Ger**	799/1729 (46)	741/1444 (51)	
	**Apartment**	295/1729 (17)	269/1444 (19)	
	**Mixed**	635/1729 (37)	434/1444 (30)	<0.001
**Household income**	**At/below minimum**	631/1549 (41)	313/1386 (23)	<0.001
	**Above minimum**	918/1549 (59)	1073/1386 (77)	
**Household member treated for tuberculosis**	**Yes**	10/1626 (1)	28/1395 (2)	0.001
	**No**	1616/1626 (99)	1367/1395 (98)	
**Breastfeeding**	**Yes**	985/1641 (60)	715/1446 (51)	<0.001
	**No**	656/1641 (40)	731/1446 (49)	
**Asthma**	**Yes**	172/1635 (11)	131/1430 (9)	0.21
	**No**	1463/1635 (89)	1299/1430 (91)	
**Length of hospital stay (days)**	**< = 7**	1939/2363 (82)	1405/1953 (72)	<0.001
	**>7**	424/2363 (18)	548/1953 (28)	
**Any previous admission**	**Yes**	758/1624 (47)	631/1437 (44)	0.13
	**No**	866/1624 (53)	806/1437 (56)	
**Antibiotic given in hospital**	**Yes**	1876/1994 (94)	1484/1624 (91)	0.002
	**No**	118/1994 (6)	140/1624 (9)	
**O2 supplementation**	**Yes**	579/2314 (25)	526/1862 (28)	0.02
	**No**	1735/2314 (75)	1336/1862 (72)	
**Hypoxia (O2 saturation <90%)**	**Yes**	431/2156 (20)	448/1717 (26)	<0.001
	**No**	1725/2156 (80)	1269/1717 (74)	
**Severe pneumonia**	**Yes**	1443/2364 (61)	1158/1954 (59)	0.24
	**No**	921/2364 (39)	796/1954 (41)	
**Very severe pneumonia**	**Yes**	527/2364 (22)	584/1954 (30)	<0.001
	**No**	1837/2364 (78)	1370/1954 (70)	
**Primary endpoint pneumonia**	**Yes**	308/1521 (20)	175/1282 (14)	<0.001
	**No**	1213/1521 (80)	1107/1282 (86)	

a SK = Songinokhairkhan District, SB = Sükhbaatar District, BZ = Bayanzürkh District, CHD = Chingeltei District.

b Cold season refers to the winter months (Nov-March) and warm season refers to non-winter months.

c Khoros are sub-districts that are categorized according to the predominant housing type, either ger (traditional Mongolian housing), apartment or mixed (ger and apartments).

### Primary endpoint pneumonia

**Fig 2:**
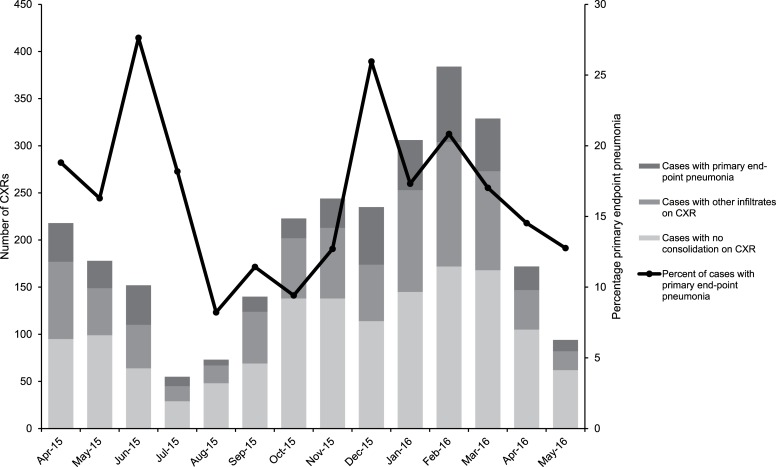
Radiology results and proportion of chest X-rays showing primary endpoint pneumonia by month for children 2–59 months, Mongolia, April 2015-May 2016 (n = 2803).

A total of 2878 (67%) of the enrolled children had CXR results over the baseline period and only 75 (3%) of these were uninterpretable. Of the interpretable CXRs (n = 2803), 483 (17%) showed PEP, either alveolar consolidation or pleural effusions, 874 (31%) showed other infiltrates and 1446 (52%) showed no consolidation. The proportion of PEP, combined across age groups and sites, varied across the baseline period between 8% and 28% (Fig 2) with the highest proportions observed in June 2015 (summer) and December 2015 (winter).

Characteristics and risk factors were compared between pneumonia cases who had PEP detected on CXR and those who had normal CXRs with no abnormalities detected as these were the two most reliable endpoints (S1 Table). Differences were found by district, with children with pneumonia from Sukhbaatar (aOR 2.01, 95% CI 1.41–2.87), Songinokhairkhan (aOR 3.66, 95% CI 2.65–5.07) and Chingeltei (aOR 2.24, 95% CI 1.48–3.40) all more likely to have PEP on CXR than those from Bayanzürkh. Children with a hospital stay of less than 7 days (aOR 0.57, 95% CI 0.43–0.75) were less likely to have PEP on their CXRs than those with longer stays. CXRs were more likely to show PEP in children from crowded households (aOR 1.35, 95% CI 1.04–1.77) with siblings (aOR 1.36, 95% CI 1.05–1.77), in children with a history of a previous admission in the last 10 days (aOR 1.68, 95% CI 1.15–2.45), in children with malnutrition (aOR 2.30, 95% CI 1.40–3.79) and in children with very severe pneumonia (aOR 1.76, 95% CI 1.35–2.30).

### Sensitivity analysis

CXRs were available for 67% (2878/4318) of children. On sensitivity analysis, using imputed case numbers for PEP incidence rates (S2 Table), the trend towards a higher incidence of PEP hospitalizations in district 1 hospitals remained in all age groups. Using multiple imputation, the incidence for children in all districts aged 2–11 months (12.0 [95% CI 10.6–13.6] per 1000 population) was nearly four-fold higher than that for children aged 12–59 months (3.3 [95% CI 3.0–3.6] per 1000 population).

## Discussion

This study describes results from a large series of pneumonia cases among children aged 2–59 months in Ulaanbaatar, Mongolia, prior to PCV13 introduction. Rates of pneumonia were higher in young children (aged 2–11 months) compared with older children (aged 12–59 months) and generally higher in the Songinokhairkhan and Sükhbaatar (“Phase 1”) Districts compared with the other two districts. In addition children in phase 1 districts were more likely to have certain risk factors for pneumonia, including more siblings, exposure to coal for cooking, cigarette smoke in the home and a lower household income, than phase 2 districts.

Pneumonia in Mongolia is highly seasonal and increases in hospital admissions usually correspond to the winter season. Severe acute respiratory infection (SARI) surveillance in Mongolia demonstrated peaks in SARI cases between October and December and between January and March [12]. In 2015/2016 the coldest months were from December 2015 to February 2016. In our study we observed an increase in pneumonia hospitalizations in February and March 2016 which also corresponded to an increase in circulating respiratory syncytial virus detected by study testing, as well as influenza virus detected by routine surveillance in Mongolia [13].

The incidence of all-cause pneumonia for children 2–59 months was 31.8 per 1000 population while that for severe pneumonia was 19.2 per 1000 population. The severe pneumonia estimates are similar to those reported by a 2015 global burden model for Mongolia which reported an incidence of 21 (8‐51) per 1000 in children <5 years of age [14]. Our estimate of 3.6 (3.2–3.9) per 1000 population for PEP is similar to that found in a Fiji study (4.3 (3.5–5.3) per 1000). The Fiji study was also conducted prior to PCV introduction and included children from 1–59 months of age [15]. Younger children aged 2–11 months had a much higher incidence of pneumonia which may be due to a higher burden of disease, but also due to the fact that younger children are more likely to be admitted.

A randomized vaccine-probe trial from Indonesia identified 28,503 cases of clinical pneumonia; 3308 were severe pneumonia cases that were hospitalized and of the 3171 that had CXRs, 22% were radiologically-confirmed based on the WHO case definition [16]. A study from the Pacific region showed that 34% of children admitted with pneumonia had PEP [15]. In our study 17% of children with CXRs had PEP. The proportion of PEP varied across the baseline period (between 8% and 28%) and didn’t seem directly related to season. These variations may be related to other factors such as differences in admission criteria and bed availability at different times of the year, as PEP cases differed from cases with normal CXR in terms of district and disease severity.

Several studies have suggested that bacterial pneumonia cannot be differentiated from non-bacterial pneumonia on the basis of the chest radiograph. However some studies have shown that bacterial, especially pneumococcal infections, are more frequently detected in patients with radiologically confirmed pneumonia compared with normal CXRs [17–19].

There were some baseline differences in the characteristics of children enrolled in the phase 1 and phase 2 districts of Ulaanbaatar. These differences included both socio-economic factors, such as housing type and crowding, as well as clinical presentation, antibiotic prescription and oxygen delivery. It will be important to adjust for district-level differences when we assess the impact of PCV13 in this setting. A previous study exploring risk factors for lower respiratory tract infections in rural Mongolia [20] found that smoking in the home, low birthweight and being a male child increased the likelihood of pneumonia admission while exclusive breastfeeding for more than 4 months and an unwillingness to seek healthcare was associated with a lower likelihood of admission.

Our surveillance program had a number of strengths. The program is population-based and includes a large number of children, which provides robust baseline estimates against which to measure PCV impact in Mongolia. Most people in Ulaanbaatar access healthcare in the public sector so our surveillance is likely representative of the general population. The Mongolian MOH chose to introduce PCV in a graded fashion into Ulaanbaatar, which will enable us to compare vaccinated and unvaccinated populations concurrently, rather than only pre- and post-PCV comparisons.

There were a number of limitations in our study. Firstly, a number of cases were missed in 2015 when the surveillance program was first started. Cases that were missed were enrolled retrospectively. Even though good medical records were available, there was some missing data for these cases and CXRs were usually not available for these children. When we imputed missing data, however, our primary findings did not change overall. The results without imputation are likely an underestimate; additional “PEP” cases are added with the imputation so all incidence estimates are increased. Secondly, during study start-up and implementation the updated WHO case definition, which excluded chest indrawing as a severe sign for pneumonia hospitalization, was introduced into hospitals in Ulaanbaatar. For the surveillance program however we used a standard case definition for enrolment and set definitions for severe and very severe pneumonia. In addition our primary study endpoint was PEP. Our enrolment case definition was an adapted WHO case definition and hospital doctors indicated that they still admitted children with chest indrawing after the WHO case definition changed. It is therefore unlikely that this change would have influenced our case numbers significantly. Thirdly, our incidence calculations may be an underestimation of case numbers as only some cases will present to health facilities and be admitted. However, in Mongolia, hospital care for children is free of charge and there is a low threshold for admission of children, so it is unlikely that severe cases were missed unless they died prior to admission. In addition given the urban setting of the evaluation we consider it unlikely that geographical barriers prevented health care seeking. However, we may have underestimated very severe pneumonia incidence rates as oxygen therapy was considered to be a proxy for ICU admissions which were not directly recorded.

In conclusion we have shown a high burden of pneumonia in children <5 years of age in Mongolia prior to the introduction of PCV. Rates differed somewhat by district and age group and were influenced by a number of socio-economic factors. It will be important to consider these differences and risk factors when assessing the impact of PCV post-introduction.

## Supporting information

S1 Text Supplementary methods: Epidemiology of pneumonia in the pre-pneumococcal conjugate vaccine era in children <5 years of age, in Ulaanbaatar, Mongolia, 2015–2016. (DOCX)

S1 Table Comparison of children 2–59 months of age with primary endpoint pneumonia and negative chest X-ray findings, living in all four districts of Ulaanbaatar, prior to PCV13 introduction in Mongolia, April 2015 to May 2016 (n = 4318). (DOCX)

S2 Table Multiple imputation estimates of incidence rates for primary endpoint pneumonia by category, age and district, in children 2–59 months of age in Mongolia, April 2015-May 2016 (per 1000 population). (DOCX)
