# Supplementary material for: Epidemiology of pneumonia in the pre-pneumococcal conjugate vaccine era in children 2-59 months of age, in Ulaanbaatar, Mongolia, 2015-2016
Source: PLoS One. 2019 Sep 11;14(9):e0222423. doi: 10.1371/journal.pone.0222423 (PMC6738602; doi:10.1371/journal.pone.0222423)
Supplement: S1 Text — (DOCX) [file pone.0222423.s001.docx]

**Supplementary methods**

**Epidemiology of pneumonia in the pre-pneumococcal conjugate vaccine era in children <5 years of age, in Ulaanbaatar, Mongolia, 2015-2016**

During initiation of the enhanced pneumonia surveillance program in 2015, some eligible pneumonia cases were missed and not enrolled prospectively into the program. A retrospective review was therefore conducted of all pneumonia admissions in the first year of surveillance to ensure that all eligible missed cases were captured. Case report forms were completed for these cases using hospital medical records. Chest x-rays (CXRs) were however missing for these retrospectively enrolled cases. To determine if there were any systematic differences we compared children with and without CXRs, and noted some differences by district and season.

We compared characteristics and risk factors between pneumonia cases who had WHO-defined primary endpoint pneumonia detected on CXR and those with normal CXR using logistic regression.

Multiple imputation was used to estimate CXR results, namely the presence or absence of primary endpoint pneumonia, for the cases who did not have CXRs done. The predictors for the multiple imputation model were based on those factors that differed significantly between patients with and without positive CXR findings on multivariable analysis. These factors included district, siblings, oxygen supplementation, malnutrition, length of hospital stay and previous recent hospital admission (Table S1). In addition we also included auxiliary variables which could potentially affect CXR taking behaviour, such as age. We used multivariate imputation by chained equation (MICE) and generated 50 imputations. Results from the 50 imputed datasets were combined using Rubin’s rules [1].

We recalculated incidence rates using imputed case numbers (Table S2). The incidence rates using imputed case numbers showed similar patterns to those using unimputed case numbers. The point estimates were higher overall, although some of the 95% confidence intervals overlapped with original estimates. The highest incidence rates were seen in children 2-11 months and in phase I districts.

**References**

1. Rubin DB. Multiple Imputation for Nonresponse in Surveys. John Wiley & Sons Inc. New York 1987.
