# Supplementary material for: Epidemiology of pneumonia in the pre-pneumococcal conjugate vaccine era in children 2-59 months of age, in Ulaanbaatar, Mongolia, 2015-2016
Source: PLoS One. 2019 Sep 11;14(9):e0222423. doi: 10.1371/journal.pone.0222423 (PMC6738602; doi:10.1371/journal.pone.0222423)
Supplement: S1 Table — (DOCX) [file pone.0222423.s002.docx]

**S1 Table. Comparison of children 2-59 months of age with primary endpoint pneumonia and negative chest X-ray findings, living in all four districts of Ulaanbaatar, prior to PCV13 introduction in Mongolia, April 2015 to May 2016 (n=4318)**

| **Category** | **Sub-category** | **Case numbers n/N (%)** | | | **Univariate analysis** | | **Multivariable analysis^a^** | |
| --- | --- | --- | --- | --- | --- | --- | --- | --- |
|  |  | **Children without CXR changes** | **Children with primary endpoint pneumonia** | | **Odds ratio (95% CI confidence interval)** | **p-value** | **Adjusted odds ratio (95% CI)** | **p-value** |
| **Sex** | **Female** | 629/1446 (44) | 232/483 (48) | Reference | |  |  |  |
|  | **Male** | 817/1446 (57) | 251/483 (52) | 0.83 (0.68-1.02) | | 0.08 |  |  |
| **Age** | **2-11 months** | 561/1446 (39) | 193/483 (40) | Reference | |  |  |  |
|  | **12-59 months** | 885/1446 (61) | 290/483 (60) | 0.95 (0.77-1.18) | | 0.65 |  |  |
| **District** | **Bayanzürkh** | 535/1446 (37) | 109/483 (23) | Reference | |  | Reference |  |
|  | **Chingeltei** | 186/1446 (13) | 66/483 (14) | 1.74 (1.23-2.47) | | 0.002 | 2.24 (1.48-3.40) | <0.001 |
|  | **Songinokharkhan** | 351/1446 (24) | 207/483 (43) | 2.89 (2.21-3.78) | | <0.001 | 3.66 (2.65-5.07) | <0.001 |
|  | **Sükhbaatar** | 374/1446 (26) | 101/483 (21) | 1.33 (0.98-1.79) | | 0.07 | 2.01 (1.41-2.87) | <0.001 |
| **Season** | **Warm season** | 709/1446 (49) | 202/483 (42) | Reference | |  |  |  |
|  | **Cold season** | 737/1446 (51) | 281/483 (58) | 1.34 (1.09-1.65) | | 0.006 |  |  |
| **Number of siblings** | **No siblings** | 925/1297 (71) | 284/483 (64) | Reference | |  | Reference |  |
|  | **Any siblings** | 372/1297 (29) | 160/483 (36) | 1.40 (1.12-1.76) | | 0.004 | 1.36 (1.05-1.77) | 0.02 |
| **Crowding (People/room)** | **<=3** | 950/1276 (74) | 272/483 (62) | Reference | |  | Reference |  |
|  | **>3** | 326/1276 (26) | 166/483 (38) | 1.78 (1.41-2.24) | | <0.001 | 1.35 (1.04-1.77) | 0.03 |
| **Smokers in the home** | **No** | 763/1302 (59) | 235/444 (53) | Reference | |  |  |  |
|  | **Yes** | 539/1302 (41) | 209/444 (47) | 1.26 (1.01-1.56) | | 0.04 |  |  |
| **Fuel for cooking** | **Electricity/gas** | 506/1302 (39) | 127/445 (29) | Reference | |  |  |  |
|  | **Wood/Coal** | 796/1302 (61) | 318/445 (71) | 1.59 (1.26-2.01) | | <0.001 |  |  |
| **Housing type** | **Formal** | 827/1303 (63) | 233/445 (52) | Reference | |  |  |  |
|  | **Informal** | 476/1303 (37) | 212/445 (48) | 1.58 (1.27-1.96) | | <0.001 |  |  |
| **Khoro type^b^** | **Apartment** | 238/1345 (18) | 71/452 (16) | Reference | |  |  |  |
|  | **Ger/mixed** | 1107/1345 (82) | 381/452 (84) | 1.15 (0.86-1.54) | | 0.33 |  |  |
| **Household income** | **Above minimum** | 876/1233 (71) | 257/422 (61) | Reference | |  |  |  |
|  | **At/below minimum** | 357/1233 (29) | 165/422 (39) | 1.58 (1.25-1.98) | | <0.001 |  |  |
| **Household member treated for tuberculosis** | **No** | 1269/1286 (99) | 429/433 (99) | Reference | |  |  |  |
|  | **Yes** | 17/1286 (1) | 4/433 (1) | 0.70 (0.23-2.08) | | 0.52 |  |  |
| **Asthma** | **No** | 1179/1294 (91) | 382/443 (86) | Reference | |  |  |  |
|  | **Yes** | 115/1294 (9) | 61/443 (14) | 1.64 (1.18-2.28) | | 0.004 |  |  |
| **Malnutrition** | **No** | 1357/1409 (96) | 419/472 (89) | Reference | |  | Reference |  |
|  | **Yes** | 52/1409 (4) | 53/472 (11) | 3.30 (2.22-4.91) | | <0.001 | 2.30 (1.40-3.79) | 0.001 |
| **Length of hospital stay (days)** | **>7** | 320/1446 (22) | 168/482 (35) | Reference | |  | Reference |  |
|  | **<=7** | 1126/1446 (78) | 314/482 (65) | 0.53 (0.42-0.67) | | <0.001 | 0.57 (0.43-0.75) | <0.001 |
| **Hospital admission in last 10 days** | **No** | 1261/1368 (92) | 383/444 (86) | Reference | |  | Reference |  |
|  | **Yes** | 107/1368 (8) | 61/444 (14) | 1.88 (1.34-2.62) | | <0.001 | 1.68 (1.15-2.45) | 0.007 |
| **Previous admission for pneumonia** | **No** | 867/1297 (67) | 250/441 (57) | Reference | |  |  |  |
|  | **Yes** | 430/1297 (33) | 191/441 (43) | 1.54 (1.23-1.92) | | <0.001 |  |  |
| **Antibiotic given in hospital** | **No** | 122/1378 (9) | 34/462 (7) | Reference | |  |  |  |
|  | **Yes** | 1256/1378 (91) | 428/462 (93) | 1.22 (0.82-1.82) | | 0.32 |  |  |
| **O2 supplementation^c^** | **No** | 1083/1422 (76) | 273/475 (57) | Reference | |  |  |  |
|  | **Yes** | 339/1422 (24) | 202/475 (43) | 2.36 (1.90-2.94) | | <0.001 |  |  |
| **Hypoxia (O2 saturation <90)^c^** | **No** | 1023/1277 (80) | 300/430 (70) | Reference | |  |  |  |
|  | **Yes** | 254/1277 (20) | 130/430 (30) | 1.74 (1.36-2.24) | | <0.001 |  |  |
| **Severe pneumonia** | **No** | 557/1446 (39) | 168/483 (35) | Reference | |  |  |  |
|  | **Yes** | 889/1446 (61) | 315/483 (65) | 1.17 (0.95-1.46) | | 0.14 |  |  |
| **Very severe pneumonia^c^** | **No** | 1086/1446 (75) | 297/483 (61) | Reference | |  | Reference |  |
|  | **Yes** | 360/1446 (25) | 186/483 (39) | 1.89 (1.52-2.35) | | <0.001 | 1.76 (1.35-2.30) | <0.001 |
| **Fever (>38^o^C)** | **No** | 744/1446 (51) | 251/483 (52) | Reference | |  |  |  |
|  | **Yes** | 702/1446 (49) | 232/483 (48) | 0.98 (0.80-1.20) | | 0.85 |  |  |
| **Respiratory rate (>50 breaths/min)** | **No** | 462/1446 (32) | 146/483 (30) | Reference | |  |  |  |
|  | **Yes** | 984/1446 (68) | 337/483 (70) | 1.08 (0.87-1.36) | | 0.48 |  |  |

^a^ Only variables significant in final multivariable model are shown.

^b^ Khoros are sub-districts that are categorized according to the predominant housing type, either ger (traditional Mongolian housing), apartment or mixed (ger and apartments).

^c^ Oxygen supplementation, hypoxia and very severe pneumonia were all significantly associated (p<0.001) and only one remained significant in different permutations of the final model. We retained very severe pneumonia in the final model as this variable included a higher number of observations compared with the other two variables.
