# Supplementary material for: Epidemiology of pneumonia in the pre-pneumococcal conjugate vaccine era in children 2-59 months of age, in Ulaanbaatar, Mongolia, 2015-2016
Source: PLoS One. 2019 Sep 11;14(9):e0222423. doi: 10.1371/journal.pone.0222423 (PMC6738602; doi:10.1371/journal.pone.0222423)
Supplement: S2 Table — (DOCX) [file pone.0222423.s003.docx]

**S2 Table. Multiple imputation estimates of incidence rates for primary endpoint pneumonia by category, age and district, in children 2-59 months of age in Mongolia, April 2015-May 2016 (per 1000 population)**

|  | **Incidence rate (95% CI)**  **All districts** | **Incidence rate (95% CI)**  **Phase 1 districts** | **Incidence rate (95% CI)**  **Phase 2 districts** | **Incidence rate (95% CI)**  **Songinokhairkhan District** | **Incidence rate (95% CI)**  **Sükhbaatar District** | **Incidence rate (95% CI) Bayanzürkh District** | **Incidence rate (95% CI) Chingeltei District** |
| --- | --- | --- | --- | --- | --- | --- | --- |
| **2-11 months** | 12.0 (10.6-13.6) | 15.0 (12.8-17.6) | 9.0 (7.3-11.0) | 15.5 (12.8-18.5) | 13.9 (9.9-19.0) | 10.6 (8.2-13.3) | 6.1 (3.8-9.2) |
| **12-59 months** | 3.3 (3.0-3.6) | 4.3 (3.8-4.9) | 2.3 (1.9-2.7) | 3.8 (3.2-4.5) | 5.5 (4.5-6.8) | 1.8 (1.4-2.2) | 3.4 (2.7-4.4) |
| **2-59 months** | 4.6 (4.3-5.0) | 6.0 (5.4-6.6) | 3.3 (2.9-3.8) | 5.7 (5.0-6.4) | 6.7 (5.6-8.0) | 3.0 (2.6-3.6) | 3.9 (3.1-4.8) |

CI=confidence interval; Phase 1 districts=Songinokhairkhan District and Sükhbaatar District; Phase 2 Districts= Bayanzürkh District and Chingeltei District
